# The Model of Occupational Self‐Efficacy: A Contemporary Model to Enhance Work Skills, Cognitive and Emotional Skills of Individuals With Brain Injury—A Pilot Randomized Control Trial

**DOI:** 10.1155/oti/5561541

**Published:** 2026-04-01

**Authors:** Mogammad Shaheed Soeker, Farhana Karachi

**Affiliations:** ^1^ Occupational Therapy Department, University of the Western Cape, Cape Town, South Africa, uwc.ac.za; ^2^ Physiotherapy Department, University of the Western Cape, Cape Town, South Africa, uwc.ac.za

**Keywords:** anxiety, cognitive skills, depression, occupational therapy, traumatic brain injury, work skills

## Abstract

**Background:**

Returning to work for survivors of traumatic brain injury (TBI) remains problematic despite them completing treatment programs.

**Objective:**

The aim of the study was to determine whether the work skills and general functioning of brain injury survivors improve after participating in a treatment program that utilizes the Model of Occupational Self‐Efficacy (MOOSE) as a framework compared with usual treatment.

**Participants:**

Twenty‐eight brain injury survivors with a mild to severe brain injury engaged in the study.

**Method:**

A randomized control design was used in the study. The Work Rehabilitation Questionnaire (WORQ), Beck Anxiety Inventory, Beck Depression Inventory, and Rowland Universal Dementia Assessment Scale (RUDAS) were used to investigate whether the work skills, cognitive functioning, and anxiety and depression symptoms of individuals improve after participating in an experimental treatment.

**Results:**

The study suggests that 56% of the individuals in the MOOSE (experimental group) had higher scores on the WORQ when compared to individuals that received usual treatment. Furthermore, 64% of individuals in the MOOSE (experimental group) had higher scores on the RUDAS when compared to individuals who received usual treatment. Regarding the Beck depression test, 64% of individuals in the MOOSE (experimental group) had a decrease in depression symptoms when compared to individuals who received usual treatment. The Beck anxiety test revealed that 57% of individuals in the MOOSE (experimental group) had a decrease in anxiety symptoms when compared to individuals who received usual treatment. The findings of the WORQ, RUDAS, Beck Depression Inventory, and Beck Anxiety Inventory were not statistically significant. Finally, 57.1% of individuals who received the MOOSE intervention returned to competitive employment compared to 28.6% of individuals who received usual treatment.

**Conclusion:**

Although the findings were not statistically significant, the experimental intervention (MOOSE) did reveal an improvement in work skills and improvement in cognitive skills as well as a reduction in symptoms of depression and anxiety for TBI survivors.

## 1. Introduction

A TBI is often linked with limitations such as impaired awareness, visual perceptual difficulties, and limitations with executive functions. In addition, individuals with TBI may have psychological limitations [1]. In South Africa, men have a higher rate of TBI than women. An estimate of 50% of TBIs are caused by cars, bikes, or pedestrian‐vehicle accidents, with the second most common cause of TBI being attributed to falls, which is more frequent among younger people [2].

As a result of the latter functional deficits that individuals with brain injury experience, they struggle to maintain employment [3]. Internationally, there is a limitation with regard to RTW for individuals with brain injury, with the RTW rate varying from 30% to 80% [4, 5].

There is a lack of clinical studies in South Africa that support the effectiveness of vocational rehabilitation. The current study explored whether the Model of Occupational Self‐Efficacy (MOOSE) is an effective model used by occupational therapists compared to usual vocational rehabilitation in reintegrating TBI individuals to the workplace.

## 2. Literature Review

Brain‐related injury is viewed as a major health and socioeconomic concern globally [6]. TBI is prevalent in low‐ and high‐income populations and influences people of all ages. In a systematic review conducted by Cancelliere et al. [7], age was associated with return to work and their study indicated that workers between the ages 20 and 29 years discontinued social benefits quicker than older aged individuals. Similarly, in O’Brien [8], age was viewed as associated with RTW, with TBI survivors under the age of 40 having a better prognosis than those over the age of 40. According to Barman et al. [9], individuals who sustained a TBI benefit from cognitive rehabilitation especially if they are well motivated and when the intervention is provided by an interdisciplinary team.

In terms of approaches to rehabilitation, peer mentoring, goal planning, engaging in physical activity, and behavioral therapy have been seen as effective for both group and individual therapy [10]. Moller et al. [11] noted that, globally, TBI survivors who suffered a severe TBI have a significantly lower probability (i.e., up to 74%) of returning to work than individuals who suffered mild and moderate TBIs.

According to McPherson [12], it is argued that the supported employment model, program‐based rehabilitation, and the case management models are the most common models used to facilitate return to work for brain injury survivors. However, it could be argued that the supported employment model is most successful to facilitate the return to employment [12].

**Figure 1:**
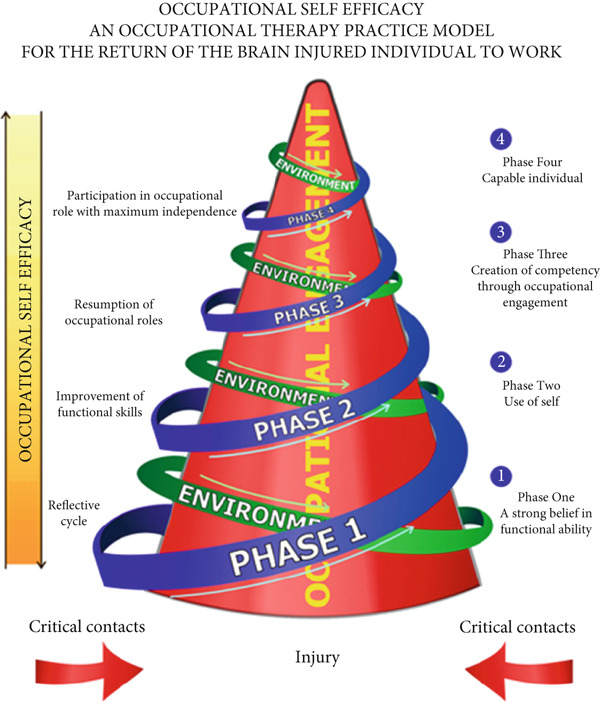
Permission was granted from the author to utilize the diagram of the MOOSE.

The MOOSE developed by Soeker [13] was initially designed to enhance the work skills of brain injury survivors. The model uses a similar framework to supportive employment; however, it is more focused on the holistic management of individuals with functional limitations (please see Figure 1).

## 3. Study Aim

The aim of the study was to determine the work skills and general functioning of brain injury survivors to improve after participating in a treatment program that utilizes the MOOSE.

## 4. Objectives

o. To investigate whether the brain injury survivors′ cognitive performance improves after participating in the MOOSE when compared to usual treatment.
o. To investigate whether the brain injury survivor′s work rehabilitation skills improve after participating in the MOOSE when compared to usual treatment.
o. To investigate whether the brain injury survivor′s level of depression improves after participating in MOOSE when compared to usual treatment.
o. To investigate whether the brain injury survivor′s level of anxiety improves after participating in MOOSE when compared to usual treatment.
o. To investigate whether the brain injury survivor′s return to work rate improves after participating in MOOSE when compared to usual treatment.

## 5. Methods

### 5.1. Research Design

Randomized research designs are seen as experimental research designs that enable a researcher to make valid conclusions with reference to cause and effect relations [14]. In the current study, an RCT design was appropriate in that it enabled the researcher to determine whether the MOOSE is an effective model compared to usual treatment in advancing the cognitive skills, work skills, and reduction in symptoms of depression and anxiety.

### 5.2. Population and Sampling

#### 5.2.1. Gaining Access to Participants

**Figure 2:**
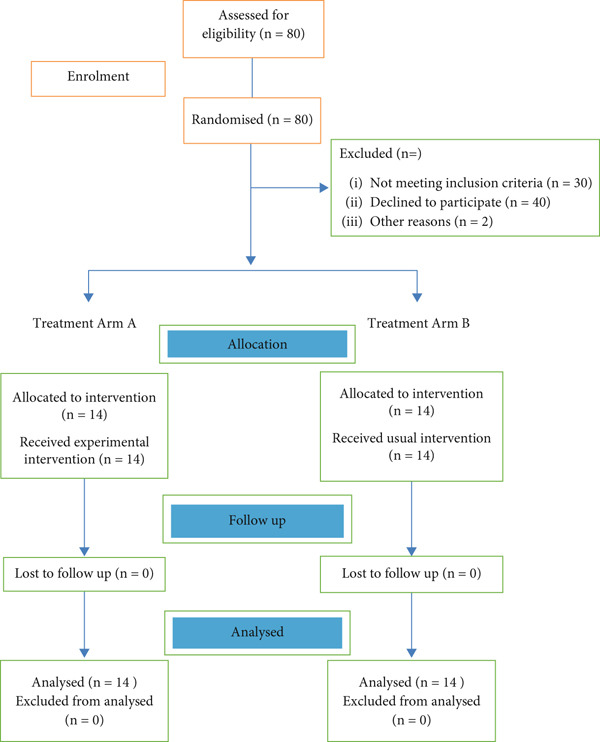
Consort diagram design of a randomized controlled trial.

Twenty‐eight (*n* = 28) participants from the statistical records of the occupational therapy departments of tertiary hospitals, namely, Tygerberg Hospital, community health centers, and one nongovernmental organization, were selected by means of simple random sampling. The researcher reviewed the statistical records of 100 participants who received treatment for their TBI in terms of eligibility to participate in the study. The researcher spent 8 months recruiting adequate participants to participate in the study. Julious [15] indicates that a sample size of 12 subjects per treatment arm is adequate in order to conduct a pilot study specifically for RCT designs. The researcher managed to recruit 14 participants in each arm of the study, i.e., 14 participants were randomly selected from the total population to participate in the experimental trial, that is, the MOOSE rehabilitation program, and 14 participants were provided with usual care or standardized care (please see Figure 2).

The study coordinator used a computer program in order to randomly select the research participants. The study coordinator blinded both the participants and occupational therapists to the two treatment groups. The coordinator (who holds a PhD) was responsible for the assignment of the research participants to the experimental group or placebo control group and was the evaluator in the study. The study coordinator did the baseline and outcome evaluations for both groups.

#### 5.2.2. Data Collection

The Work Rehabilitation Questionnaire (WORQ), Beck Anxiety Questionnaire, Beck Depression Questionnaire, and RUDAS questionnaires were administered before and after the research participants participated in intervention. The research participants were randomly assigned to either an experimental treatment group using the MOOSE as the structure for rehabilitation. The same evaluator conducted baseline and postintervention evaluations. The evaluator had undergone comprehensive training prior to the commencement of the research study. Training consists of the initial presentation of each test individually, guided practice, and individual practice with mock participants.

### 5.3. Inclusion Criteria

Participants were diagnosed with either a mild, moderate, or severe brain injury according to the Glasgow Coma Scale. They must have been employed for at least 3 months prior to the injury. The participants must be 6–36 months post TBI and must have received rehabilitation from a multidisciplinary team. Furthermore, the participants should be able to communicate effectively in English or isi Xhosa or Afrikaans and be able to understand verbal questions.

### 5.4. Exclusion Criteria

Members who had psychiatric symptoms such as delusions and hallucinations were excluded from the study as they may not be able to participate in the intervention due to their illness.

#### 5.4.1. Experimental Treatment (Experimental Group)

The participants in this research study who participated in the experimental intervention followed the rehabilitation procedure of the MOOSE [13]. The MOOSE program was administered by a qualified occupational therapist who had experience in treating individuals with TBI.

The MOOSE is described by the following stages, namely, Stage 1: “a strong personal belief in functional abilities,” the participants engaged in a reflection process using the Gibbs reflective cycle (see Table 1) [16]. In Stage 2: “use of self,” the participants participated in activities that would enhance their individual capabilities, such as improving their endurance, muscle strength, and cognitive skills. In Stage 3: “creation of competency through occupational engagement,” the brain injury survivors engaged in simulated tasks to enhance their work abilities. The brain injury survivors participated in real work tasks such as that or a cashier or storemen. The role‐plays enhanced skills important to work such as life skills, communication skills, and planning skills. In Stage 4: “capable individual” which is the final stage of the model, the brain injury survivor would engage in real work for a period of time after the completion of the program. These individuals would then continue to get ongoing support in the workplace if required with a gradual reduction in workplace support given. The type of support entailed vocational counseling or advice on work simplification methods. The duration of the program was approximately 8 weeks and clients were seen once per week, namely, two sessions on a Thursday or two sessions on a Friday. Each session was approximately 1.5 h long. The sessions were conducted at a rehabilitation center.

**Table 1:** Intervention program (usual treatment and experimental treatment).

Intervention program		
	Usual treatment	MOOSE treatment
Preintervention stage: Screening assessments completed to determine the participant′s level of functioning	• RUDAS • WORQ • Beck anxiety questionnaire • Beck depression questionnaire	• RUDAS • WORQ • Beck anxiety questionnaire • Beck depression questionnaire
Stage 1: Weeks 1–2	The intervention in this stage is geared at improving attention, orientation, and insight Type of activities used: • Verbal orientation questions and making of orientation board or poster • Practical ways to improve attention: Synonym and antonym worksheets, thinking skills questionnaire, rearrange the sentence worksheet, Thurstone questionnaire • Level of insight with regard to functional and vocational capabilities	“A strong personal belief in functional abilities” The therapist uses reflection and introspection to facilitate insight into the participant′s perception of their vocational capabilities Type of activities: Reflective journal, creative writing, individual and group sessions with clients Group activity using Gibbs reflective cycle 1. What happened 2. What were you thinking and feeling 3. What was good and bad about the experience 4. What sense can you make of the situation 5. What else could you have done 6. If the situation arose again, what would you do
Stage 2: Weeks 3–4	This stage focuses on improving memory and executive function skills: Type of activities: Using external aids to improve memory, how to remember a picture Executive function activities: Plan your busy morning activity, rearrange the pictures in order of priority	“Use of self” Use of activities that will improve the client′s memory, concentration, and executive function Type of activities: Crossword puzzles, Kim′s game, tabletop activities, reading newspaper articles, and writing down information
Stage 3: Weeks 5–6	Vocational exploration Vocational exploration for participants who are currently unemployed, that is, learnership, recruitment agencies for persons with disabilities	“Creation of competency through occupational engagement” During this stage, the participants will engage in tasks related to a specific job to which they want to return (e.g., packer, cleaner, and administrator) Type of activities: Role‐play (to improve coping skills, problem‐solving skills, and communication skills) Actual work test placement: Support will be provided for improvement of work skills and work habits
Stage 4: Weeks 7–8	No activity	“Capable individual” The participants resume their current jobs or seek alternative employment in the open labor market. Participants will continue to receive support if necessary; however, the amount of support is gradually reduced to promote functional independence
Post vocational stage: To determine outcome of cognitive skills after completion of intervention program	• RUDAS • WORQ • Beck anxiety questionnaire • Beck depression questionnaire	• RUDAS • WORQ • Beck anxiety questionnaire • Beck depression questionnaire

#### 5.4.2. Usual Treatment (Control Group)

The participants in this research study who participated in the usual treatment (placebo) intervention followed a standardized treatment protocol. The usual treatment program was administered by a qualified occupational therapist who had experience in treating individuals with TBI. The standardized protocol is described as follows: Weeks 1–2: Individuals participated on improving the client′s attention and insight in the brain injury; Week 3–4: Focus was on improving the TBI survivor′s memory ; Week 5–6: Focus is on identifying suitable work opportunities. The duration of the program was approximately 6 weeks, and clients were seen once per week. Each session was approximately 2–3 h long. The sessions were conducted at a rehabilitation center.

### 5.5. Reliability and Validity

In the current study, the questionnaire was piloted with four participants; the participants responded appropriately to the questionnaire. The questionnaires used were the ones described below.

#### 5.5.1. Beck Depression Inventory (BDI‐II)

This is a 21‐item self‐report instrument that is aimed at measuring the severity of depression in adults [17]. It was developed to assess the symptoms that corresponded to the diagnosis of depressive disorders as indicated in the Diagnostic and Statistical Manual of Mental Disorder, fourth edition (DSM‐IV). The BDI‐II builds on 35 years of accumulated data collection both in psychometric and clinical aspects and has proved to be valid and reliable [17]. A total score of 0–7 is considered minimal range of depression, 8–15 is mild depression, 16–25 is moderate depression, and 26–63 is severe depression. An increase in the scores on the BDI‐II is an indicator of an increase in symptoms of depression.

#### 5.5.2. Beck Anxiety Inventory (BAI)

According to Beck and Steer [17], the BAI was developed to determine an individual′s symptoms of anxiety. This is a self‐report instrument that has been found reliable for adults (i.e., over the age of 17 years), for the detection of symptoms of anxiety. Scores may range from 0 to 63: minimal anxiety levels (0–7), mild anxiety (8–15), moderate anxiety (16–25), and severe anxiety (26–63). An increase in the scores on the BAI is an indicator of an increase in symptoms of anxiety.

#### 5.5.3. WORQ

This instrument was designed to better understand the limited functional capability of individuals as a result of a medical condition [18]. According to Finger et al. [18], it was found that WORQ has high test reliability and good internal consistency. An increase in the scores on the WORQ is an indicator of a positive improvement in work‐related skills.

#### 5.5.4. RUDAS

The RUDAS questionnaire is a six‐item questionnaire that assesses multiple cognitive domains such as memory, praxis, language, judgment, drawing, and body orientation. The test showed adequate validity and reliability [19]. The RUDAS questionnaire has a cutoff score of 22 or less (lower scores indicate greater cognitive impairment) and 23–30 considered normal; however, scores need to be considered in the clinical context.

NB: Demographic information and medical information were obtained from the client′s medical records at a tertiary hospital in Cape Town.

### 5.6. Data Analysis

Descriptive and inferential data were summarized in tables for analysis. In the study, the measure of frequency and central tendency was used to describe the descriptive data from the study. An independent *t*‐test and effect size analysis were used to determine whether there was a significant difference between the means of the experimental and control groups.

## 6. Results

In Table 2, the characteristics of the 14 participants who formed part of the control group [*n* = 14] and experimental group [*n* = 14] are described. The table indicates that the participants were similar in age, level of education, severity of the brain injury, and employment status before the injury.

**Table 2:** Demographics of participants.

	**Control (mean and SD)**	**Experimental (mean and SD)**
Age	38 (SD = 9.5)	34 (SD = 6.8)
	**Control (%)**	**Experimental (%)**

Sex	Male (71%) Female (29%)	Male (93%) Female (7%)
Education	Primary (14%) Secondary (64%) Tertiary (21%)	Primary (14%) Secondary (64%) Tertiary (21%)
TBI	Mild (14%) Moderate (79%) Severe (7%)	Mild (14%) Moderate (71%) Severe (14%)
Preinjury employment	Not employed (71%) Employed (0) Volunteer (7%) Student (0) Self‐employed (7%)	Not employed (36%) Employed (21%) Volunteer (7%) Student (36%) Self‐employed (0)

*Note:* Sex: 1—female and 2—male. Education: 3—primary school, 4—secondary school, and 5—tertiary. Type of injury: 1—mild, 2—moderate, and 3—severe TBI. Status of work: 7—not employed, 1—employed, 3—volunteer, 4—student or training, and 2—self‐employed.

**Figure 3:**
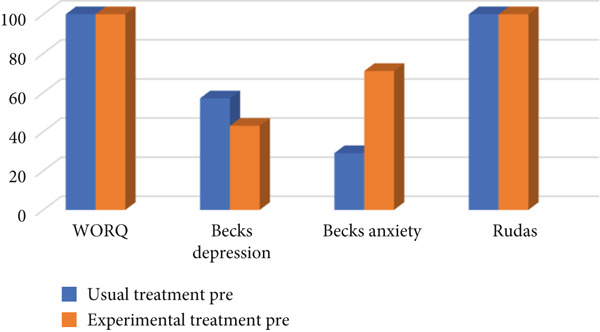
Preintervention: usual treatment versus experimental treatment.

**Figure 4:**
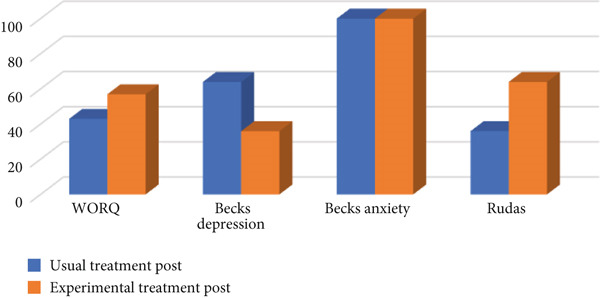
Postintervention: usual treatment versus experimental treatment.

Figures 3 and 4 describe the total scores of the participants in relation to comparing the usual treatment to experimental treatment (i.e., MOOSE) per assessment (WORQ, RUDAS, BDI, and BAI).

Table 3 describes the inferential statistics related to the mean, level of significance, *t* statistic, and effect size.

**Table 3:** Table describing mean, level of significance, and effect size (postintervention).

**Outcome measure**		**N**	**Mean**	**Standard deviation**	**Level of significance**	**t** **statistic**	**Cohen′s** **d**
WORQ	NT	14	85.6	43.1	0.35	−0.66	−0.25
	MOOSE	14	96.0	39.6			
RUDAS	NT	14	25.8	2.8	0.58	−0.40	−0.15
	MOOSE	14	26.3	3.7			
Beck depression	NT	14	17.6	10.8	0.40	1.18	0.45
	MOOSE	14	13.1	9.6			
Beck anxiety	NT	14	15.9	11.2	0.06	0.68	0.26
	MOOSE	14	13.5	6.7			

### 6.1. Pre and Posttesting

In this section, the pre and posttest results of the participants in both the control and experimental groups will be presented.

#### 6.1.1. WORQ (Pretest)

As seen in Figure 3, individuals in both control and experimental groups achieved a mean of 107. Individuals in both the experimental and control groups had similar scores with regard to the WORQ questionnaire.

#### 6.1.2. WORQ (Posttest)

As seen in Figure 4, 57% of individuals had an increase in WORQ score using MOOSE (experimental treatment) when compared with usual treatment. However, the result was not statistically significant [*t* (−0.66) = 26, *p* = 0.35]. The mean score for usual treatment was 86 and for MOOSE (experimental treatment) was 96. The latter indicates that from a descriptive perspective, the research participants rated an improvement with regard to their work‐related skills in the experimental group when compared to usual care.

#### 6.1.3. RUDAS (Pretest)

As seen in Figure 3, individuals in both groups achieved a similar mean score; the control group had a mean of 26 and the experimental group had a mean of 24. Individuals in both the experimental and control groups had similar scores with regard to the RUDAS questionnaire.

#### 6.1.4. RUDAS (Posttest)

As seen in Figure 4, there was a similar increase in scores for both individuals using the MOOSE (experimental treatment), mean being 26 and usual treatment mean being 26. Sixty‐four percent of individuals had an increase in scores on the RUDAS test when compared to usual treatment; however, the result was not statistically significant [*t* (−0.40) = 26, *p* = 0.58]. The latter indicates that from a descriptive perspective there was an improvement with regard to the TBI survivors′ cognitive skills who participated in the experimental group when compared to usual care.

#### 6.1.5. Beck Depression Test (Pretest)

As seen in Figure 3, 57% of individuals using usual care had higher scores on the depression inventory when compared to individuals receiving the MOOSE (experimental treatment). The mean score for usual treatment was 23 and for MOOSE (experimental treatment) was 21.

#### 6.1.6. Beck Depression Test (Posttest)

As seen in Figure 4, 64% of individuals using the MOOSE presented with lower depression scores when compared to individuals receiving usual treatment. However, the result was not statistically significant [*t* (1.18) = 26, *p* = 0.40]. The mean score for usual treatment was 17 and for MOOSE (experimental treatment) was 13. The latter indicates that from a descriptive perspective there was a decrease with regard to the TBI survivors′ symptoms related to depression who participated in the experimental group when compared to usual care.

#### 6.1.7. Beck Anxiety Test (Pretest)

As seen in the graph, there was an increase in the anxiety score for individuals using the MOOSE (experimental treatment). There was a 71% increase in individuals using MOOSE who had higher scores on the Beck anxiety test.

#### 6.1.8. Beck Anxiety Test (Posttest)

As seen in Figure 4, the mean score for usual treatment was 15 and for MOOSE (experimental treatment) being 13 does indicate that, in general, the symptoms of severe anxiety were less in the experimental group (i.e., severe anxiety scores ranging from 26 to 63).

In Table 4, return to work after the completion of rehabilitation program.

**Table 4:** Employment status postintervention.

**Employment outcome (%)**	**Usual treatment (control) (** **n** = 14 **)**	**MOOSE intervention (experimental) (** **n** = 14 **)**
Not employed	57.1%	21.4%
Employed (competitive employment)	28.6%	57.1%
Self‐employed	7.1%	0%
Volunteer	7.1%	7.1%
Student or training	0%	14.3%
Total	100%	100%

In the table, the following was recorded in terms of returning to employment in the labor market: the majority of the participants, 8/14 (57%), returned to work in open labor market and that 3/14 (21%) were unemployed at the time of the completion of the program.

Similarly, for individuals that completed the usual treatment program, the majority of the participants were unemployed, 8/14 (57%), at the time of the completion of the program and 4/14 (28.6%) returned to work in the open labor market. The latter results indicate that a larger percentage of individuals who participated in the experimental group were able to find employment in the open labor market/competitive employment. A possible justification could be that the MOOSE programs specifically focus on work test placement and exposure to working in real work in the open labor market whereas the control group does not focus on work test placement.

## 7. Discussion

The current study indicated that the participants had performed better cognitively on the RUDAS test using the MOOSE program. Furthermore, the participant using the MOOSE program had a reduction in depression and anxiety symptoms when compared to participants who received usual treatment. This pilot study, while not demonstrating significantly different outcomes, did indicate some positive trends in work‐related characteristics and mood disorders.

The participants′ score in the experimental group (MOOSE intervention) regarding the RUDAS cognitive assessment revealed that they obtained higher scores than the participants who were exposed to usual care, thus indicating that the MOOSE intervention may have contributed to the cognitive functioning of brain injury survivors. In a study conducted by Pruscino et al. [20], they reveal that cognitive retraining is essential to vocational rehabilitation programs.

The participant′s WORQ baseline test mean scores were similar; however, the postintervention test scores indicated that the mean of the experimental group (MOOSE) was higher than that of the control group. A reason for the latter result could be that the MOOSE program has a practical component that enables the participant to apply work‐related skills in a real work setting, thereby enhancing their confidence. According to Trexler and Parrot [21], they state that it is seldom that the work skills of individuals with TBI who do not participate in rehabilitation programs improve. Furthermore, in a cross‐sectional study conducted by Olaoye et al. [22], they reveal that individuals diagnosed with a stroke who do not participate in rehabilitation programs that enhance their work skills may have a reduced return to work rate and hence reduced WORQ scores.

The score on the BDI‐II revealed that the research participants who participated in the experimental research (MOOSE) had a decrease in depression scores when compared to usual treatment (control groups). According to Bodnar et al. [23], depression symptoms are common symptoms experienced by individuals who sustained a TBI. Depression symptoms are usually a common focus in rehabilitation for the purpose of enhancing the quality of life and worker roles of individuals with TBI [24]. In a study conducted by Mokhtari et al. [25] and Mirseify Fard and Goli [26], they found that interventions that included life skills and cognitive rehabilitation reduced the depression symptoms of research participants. In the current study, the fact that the experimental treatment mainly focused on self‐reflection, goal setting, and life skills such as coping skills, stress management, and relaxation techniques could be seen as a possible reason why individuals in the experimental group (MOOSE) had fewer depressive symptoms than individuals that received usual care. The above indicates that the research participants may benefit clinically from the MOOSE program.

The BAI indicated that individuals who participated in the experimental program (MOOSE) did reveal a decrease in general symptoms of severe anxiety when compared with usual care, post intervention. According to Al‐Kader [27], anxiety is a common symptom experienced by individuals with TBI. The anxiety symptoms often manifest as the individuals with TBI realize that they have functional limitations that affect their relationships with others, their ability to integrate into the community, and their worker roles.

The majority of individuals who participated in the study received a disability grant (a disability grant is a monthly financial stipend that is given to individuals diagnosed with a medical condition that renders them incapable of finding employment). The fact that individuals may fear losing the disability grant could be seen as a barrier for returning to work [28]. However, in the current study, the participants seemed to be motivated to find employment in the open labor market in order to earn a competitive salary. Individuals in both the experimental and control groups completed the various rehabilitation programs that were identified.

## 8. Limitations of the Study

The findings of this study cannot be generalized to a larger population due to the small sample size. Other limitations included the selection of mainly male participants. Finally, the outcome of the assessment was not blinded and this could have affected the results.

## 9. Conclusion

The current study is novel in that it is the first study that used a RCT design to study the effectiveness of MOOSE as a treatment for returning individuals with TBI to work. Return to employment is an important occupation for both the patient and society. This study, while not statistically significant, did present with data that reveal an improvement in work skills and mood compared to usual care. Although the sample size is small, the descriptive statistics do suggest that the experimental (MOOSE) program may be a useful program in enhancing work skills, improving cognitive skills, and reducing the symptoms of depression and anxiety in individuals diagnosed with TBI (mild, moderate, and severe) when compared with usual treatment. It is suggested that the study be continued with a larger sample of research participants; this may ultimately enhance the statistical significance of the study and allow for identification of who might best benefit from this type of intervention.

## Ethics Statement

The study was approved by the Biomedical Research Ethics Committee from the University of the Western Cape, ethics approval number being BM21/9/3. The randomized control trial was registered on the National Health Research Database as a RCT, the registration number being WC_201903_024.

## Consent

All participants were fully briefed about the purpose, procedures, potential risks, and benefits of the study before their involvement. A detailed participant information sheet was provided in plain language, and the consent process included one‐on‐one discussions to ensure comprehension. Participants were informed of their right to withdraw at any stage without repercussions. Written consent was obtained from each participant before any data collection commenced.

## Conflicts of Interest

The authors declare no conflicts of interest.

## Funding

The study is supported by the South African Medical Research Council.

## General Statement

*Confidentiality and Data Security*. Confidentiality was maintained throughout the research process. Participant identifiers were removed from all data records, which were instead coded to preserve anonymity. Digital data were stored on encrypted devices, accessible only to the research team. Hard copy documents, such as consent forms, were stored securely in locked cabinets. Furthermore, data were shared only in aggregated form in publications or presentations, ensuring no individual participant could be identified.

## Data Availability

The data that support the findings of this study are available on request from the corresponding author. The data are not publicly available due to privacy or ethical restrictions.
